# A Multiplex Assay for the Diagnosis of Mucopolysaccharidoses and Mucolipidoses

**DOI:** 10.1371/journal.pone.0138622

**Published:** 2015-09-25

**Authors:** Eveline J. Langereis, Tom Wagemans, Wim Kulik, Dirk J. Lefeber, Henk van Lenthe, Esmee Oussoren, Ans T. van der Ploeg, George J. Ruijter, Ron A. Wevers, Frits A. Wijburg, Naomi van Vlies

**Affiliations:** 1 Department of Pediatric Metabolic Diseases, Emma Children’s Hospital and Amsterdam Lysosome Center ‘Sphinx’, Academic Medical Center, University of Amsterdam, Amsterdam, The Netherlands; 2 Laboratory for Genetic Metabolic Diseases, Academic Medical Center, University of Amsterdam, Amsterdam, The Netherlands; 3 Translational Metabolic Laboratory, Departments of Neurology & Laboratory Medicine, Radboud University Medical Centre, Nijmegen, The Netherlands; 4 Department of Pediatrics, Center for Lysosomal and Metabolic Diseases, Erasmus Medical Center, Rotterdam, The Netherlands; 5 Department of Clinical Genetics, Center for Lysosomal and Metabolic Diseases, Erasmus Medical Center, Rotterdam, The Netherlands; University of Patras, GREECE

## Abstract

**Introduction:**

Diagnosis of the mucopolysaccharidoses (MPSs) generally relies on an initial analysis of total glycosaminoglycan (GAG) excretion in urine. Often the dimethylmethylene blue dye-binding (DMB) assay is used, although false-negative results have been reported. We report a multiplexed diagnostic test with a high sensitivity for all MPSs and with the potential to identify patients with I-cell disease (ML II) and mucolipidosis III (ML III).

**Methods:**

Urine samples of 100 treatment naive MPS patients were collected and analyzed by the conventional DMB assay and a multiplex assay based on enzymatic digestion of heparan sulfate (HS), dermatan sulfate (DS) and keratan sulfate (KS) followed by quantification by LC-MS/MS. Specificity was calculated by analyzing urine samples from a cohort of 39 patients suspected for an inborn error of metabolism, including MPSs.

**Results:**

The MPS cohort consisted of 18 MPS I, 16 MPS II, 34 MPS III, 10 MPS IVA, 3 MPS IVB, 17 MPS VI and 2 MPS VII patients. All 100 patients were identified by the LC-MS/MS assay with typical patterns of elevation of HS, DS and KS, respectively (sensitivity 100%). DMB analysis of the urine was found to be in the normal range in 10 of the 100 patients (sensitivity 90%). Three out of the 39 patients were identified as false-positive, resulting in a specificity of the LS-MS/MS assay of 92%. For the DMB this was 97%. All three patients with MLII/MLIII had elevated GAGs in the LC-MS/MS assay while the DMB test was normal in 2 of them.

**Conclusion:**

The multiplex LC-MS/MS assay provides a robust and very sensitive assay for the diagnosis of the complete spectrum of MPSs and has the potential to identify MPS related disorders such as MLII/MLIII. Its performance is superior to that of the conventional DMB assay.

## Introduction

The mucopolysaccharidoses (MPSs) comprise a group of lysosomal storage disorders caused by a deficiency in one of the lysosomal enzymes involved in the degradation of glycosaminoglycans (GAGs) (Table 1) [1]. Through complex pathophysiological cascades, the accumulation of non-degraded GAGs results in a wide range of progressive somatic and neurological manifestations, including skeletal dysplasia, cardiorespiratory compromise, and, in some of the more severe phenotypes, cognitive decline. Mucolipidosis II and III (I-cell disease and ML III) show clinical resemblance to the MPSs and are caused by inadequate post-translational modification of lysosomal enzymes, leading to an effective deficiency of multiple enzymes (Table 1) [2].

**Table 1:** The mucopolysaccharidoses and mucolipidosis II and III.

Type	Eponym	Enzyme deficiency	Storage product*
**MPS I**	Hurler,	α-L-iduronidase	DS, HS
	Hurler/Scheie,		
	Scheie		
**MPS II**	Hunter	Iduronate-2-sulfatase	DS, HS
**MPS III A**	Sanfilippo A	Heparan-N-sulfatase	HS
**MPS III B**	Sanfilippo B	N-acteyl-α-glucosaminidase	HS
**MPS III C**	Sanfilippo C	Acetyl-CoA:α-glucosaminide N-acetyltransferase	HS
**MPS III D**	Sanfilippo D	N-acetylglucosamine 6-sulfatase	HS
**MPS IVA**	Morquio A	Galactose-6-sulfatase	KS, CS
**MPS IVB**	Morquio B	β-galactosidase	KS
**MPS VI**	Maroteaux-Lamy	N-acetylgalactosamine-4-sulfatase	DS
**MPS VII**	Sly	β-glucuronidase	DS, HS, CS
**MPS IX**	-	Hyaluronidase	Hyaluronan
**ML II**	I-cell disease	N-acetylglucosaminyl-1-phosphotransferase	GAGs, sphingolipids
**ML III**	Pseudo-Hurler polydystrophy		

* DS: Dermatan sulfate, HS: Heparan sulfate, CS: Chondroitin sulfate, KS: Keratan sulfate, GAGs: glycosaminoglycans.

When a diagnosis of MPS is considered, biochemical analysis is generally performed by quantification of urinary GAGs by a dimethylmethylene blue dye binding assay (DMB) [3,4], followed by two-dimensional electrophoresis for qualification of the type of excreted GAGs. Positive screening is followed by analysis of the relevant enzyme activity in leucocytes or cultured skin fibroblasts. However, the DMB assay may lead to false-negative results, especially in MPS IV [5], and GAG electrophoresis is relatively time-consuming and dependent on subjective interpretation.

Recent studies show that quantification of specific GAG derived disaccharides by liquid chromatography tandem-mass spectrometry (LC-MS/MS) may provide a much more sensitive method for both quantification and qualification of GAGs in urine and blood as well as in other tissues [6–9]. Preparation of the samples involves either enzymatic digestion or methanolysis in order to degrade the large GAG molecules into poly- or disaccharides, followed by LC-MS/MS quantification. LC-MS/MS assays combining quantification of urinary dermatan sulfate (DS) and heparan sulfate (HS), and in some cases chondroitin sulfate (CS), have been reported [7–9] as well as separate analysis of urinary keratan sulfate (KS) [10,11]. However, none of these reported methods covers the complete spectrum of MPSs.

Especially the diagnosis of MPS IV is hampered in previously reported multiplexed assays, as these fail to detect KS accumulation. MPS IVA may be diagnosed through CS accumulation in some patients [8]. This is, however, not suitable for the diagnosis of MPS IVB, where, based on the enzyme deficiency, KS is the only primary storage material [12]. Also, the diagnostic potential for MPS VII has not been reported.

Here we report a multiplexed assay for the detection of HS, DS and KS and we extensively evaluate its diagnostic accuracy in the complete MPS spectrum in comparison to the conventional DMB method.

## Methods

### Patients and controls

Positive controls were obtained by the collection of urinary samples of treatment naive patients with enzymatically confirmed diagnosis of MPS I, MPS II, MPS III, MPS IVA, MPSIVB, MPS VI and MPS VII, ML II and ML III (Table 2). Samples from the Erasmus Medical Center, Rotterdam; and Radboud University Medical Center, Nijmegen were send to the Academic Medical Center completely anonymized for analysis. Samples from the Academic Medical Center (AMC) were collected for routine diagnostics and the remainder was encoded and stored for further research (i.e. biobanking) with the written informed consent of parents/patients. Consent forms for biobank storage were approved by the Medical Ethics Review Committee of the AMC, Amsterdam, The Netherlands. These procedures follow the AMC Research Code on research using human materials. Care was taken to include patients across the complete phenotypic spectrum of the disease where possible. Thirty-nine urine samples of patients with a suspected inborn error of metabolism (IEM), where a DMB was performed as part of the metabolic work up, were used to assess the rate of false-positive findings for both assays. Additional data was gathered for these patients, including age, gender, presenting symptoms and eventual diagnosis if available. Sixty-one anonymized urine samples from healthy controls were analyzed to determine reference values for the LC-MS/MS assay.

**Table 2:** Patient, age range and fold-ULN for HS, DS, KS, the cumulative levels of HS, DS and KS and DMB per diagnosis.

MPS subtype	Number of patients	Age range (yrs)	Median fold ULN (range)				
			HS	DS	KS	HS, DS & KS	DMB
**MPS I**	18	0.8–32.6	12.1 (3.4–2.9)	101.8 (26.3–03.4)	0.6 (0.2–1.7)	10.8 (2.6–50.3)	4.4 (1.1–0.3)
**MPS II**	16	0.2–46.3	17.2 (10.0–23.6)	69.2 (35.3–139.4)	0.5 (0.4–1.6)	9.4 (5.3–23.6)	4.5 (1.7–8.5)
**MPS III**	34	3.3–66.9	28.3 (17.8–66.9)	1.3 (0.4–5.0)	0.5 (0.3–1.6)	12.0 (6.6–27.6)	3.1 (0.9–7.3)
**MPS IVA**	10	1.1–56.6	0.9 (0.4–1.4)	3.0 (1.0–4.6)	5.2 (2.4–14.6)	3.3 (1.6–8.2)	1.2 (0.6–2.4)
**MPS IVB**	3	21.5–51.3	1.2 (1.1–1.3)	1.3 (0.7–2.2)	6.8 (5.7–10.0)	3.9 (2.9–5.0)	0.7 (0.4–0.8)
**MPS VI**	17	0.1–57.8	0.6 (0.3–2.6)	31.3 (7.3–243.2)	0.6 (0.2–1.7)	3.0 (0.6–15.6)	1.9 (0.6–9.8)
**MPS VII**	2	21.0–21.3	1.0–5.4	1.5–2.6	0.1–0.9	0.7–3.5	0.5–0.7
**ML II**	2	0.2–0.3	0.8–2.8	1.6–5.3	1.0–1.7	0.9–2.1	0.8–1.4
**MLIII**	1	12.0	1.1	2.4	1.3	1.2	0.8

### Biochemical analyses

Total urinary GAGs were measured with the dimethylmethylene blue-binding assay, as first described by De Jong et al [3,4].

GAG-derived disaccharides in urine were analyzed in a multiplex assay. The measurement of KS derived disaccharides was incorporated into our HS and DS analysis, previously described by de Ru et al [13]. The cDNA coding for keratanase II from *Bacillus circulans* (KerII, GenScript) was synthesized and cloned into the pET22b(+)(Novagen-EMD4Biosciences, Madison, WI, USA) vector, chondroitinase B from Pedobacter heparinus was cloned into pET19b (Novagen-EMD4Biosciences, Madison, WI, USA) and expression plasmids (pET15b or pET19b) containing the cDNA for heparinase I, II, and III were a generous gift from Dr. Ding Xu (University of California, CA, USA). All enzymes were expressed as His-tagged fusion proteins in E. coli (BL21 AI, Invitrogen) in Terrific Broth medium with 8 g/L glycerol at 22°C. The enzymes were purified on HisLink Protein Purification Resin (Promega) according to the manufacturer’s protocol. The purified enzymes were dialyzed against a buffer containing 50 mM Tris (pH7.5), 10 mM CaCl2, 200 mM NaCl and 2 mM DTT. Thereafter, 126 g/L glycerol and 2 mg/mL BSA were added, and aliquots were snap-frozen in liquid nitrogen and subsequently stored at -80°C.

Before each experiment, the activity of chondroitinase B and the three heparinases were tested. Heparinase I and II activity was measured at 30°C in an incubation medium (1 mL final volume) containing 25 mM HEPES (pH7.0), 100 mM NaCl, 1 mM CaCl2, 2 mM DTT and 3–4 mIU enzyme. The reaction was started by the addition of 0.2 mg/mL heparin, and the introduction of the double bond was followed over time by monitoring the absorbance at 232 nm. The activity was calculated using an extinction coefficient of 5200 L·mol−1·cm−1. The activity of heparinase III and chondroitinase B were measured essentially as described above using de-O-sulfated heparan sulfate or dermatan sulfate as substrates, respectively. The activity of KerII was determined by analyzing recovery of spiked keratan sulfate substrate in the LC-MS/MS assay using 0.5, 1, 5, 10 and 15 uL of the KerII suspension for digestion. At 5uL and above, the recovery was 100% and therefore 10 uL was used in the final assay.

Heparan sulfate, dermatan sulfate and keratin sulfate were enzymatically digested into disaccharides in a mixture containing 100 mM NH4Ac (pH7.0), 10 mM Ca(Ac)2, 2 mM DTT, 5 mIU each of heparinase I, II, III, 50 mIU chondroitinase B, 10μl of KerII and 50 μL urine diluted to 2 mM creatinine in a final volume of 150 μL. After 2 h of incubation at 30°C, 15 μL of 150 mM EDTA (pH7.0) was added along with 125 ng of the internal standard, 4UA-2S-GlcNCOEt-6S (HD009, Iduron, Manchester, UK), and the reaction was stopped by boiling for 5 min to denature the proteins. The reaction mixture was centrifuged at 20,000×g for 5 min at room temperature. The supernatant was subsequently applied to an Amicon Ultra 30 K centrifugal filter (Millipore) and centrifuged at 14,000×g for 15 min at 25°C. The filtrate was stored at -20°C until analysis.

The disaccharides were quantified on a Waters Quattro Premier XE (tandem) mass spectrometer (Waters Corporation, Milford, MA, USA) coupled to an Acquity UPLC system (UPLC-MS/MS) with a ESI source and a capillary voltage of 3.5 kV, a source temperature of 130°C, desolvation temperature of 350°C, cone gas flow of 50 L/hr, desolvation gas flow of 900 L/hr and a collision gas pressure of 2.5*10^−3^ mbar. The disaccharides were separated on a Thermo Hypercarb HPLC column (100×2.1 mm, 5 μm). The mobile phase consisted of 10 mM NH4HCO3 (pH10), and the disaccharides were eluted with an acetonitrile gradient of 0% to 20% for 2.5 min, held at 20% for the next 2.5 min, with 2 min of equilibration at 0% before the next injection; the flow rate was 0.2 mL/min, and the total run time was 7.1min.

All disaccharides (following the nomenclature as proposed by Lawrence et al [14]) were detected and quantified in the MRM acquisition mode, using the transition m/z 378.1>175.1, cone voltage of 25 V and collision energy of 14 V for D0A0, m/z 416.1>138.0, cone voltage of 40 V and collision energy of 22 V for D0S0, m/z 458.1>97.0, cone voltage of 40 V and collision energy of 34 V for D0A6 and D2A0, m/z 496.0>416.0, cone voltage of 25 V and collision energy of 16 V for D2S0 and D0S6, m/z 458.0>299.9, cone voltage of 35 V and collision energy of 22 V for D0a4, m/z 538.0>458.0, cone voltage of 20 V and collision energy of 15 V for D0a10, m/z 462.1>361, cone voltage of 55 V and collision energy of 25 V for g0A6 and g6A6 (which could be separated by their retention time of 4.7 and 5.2 min, respectively) and m/z 472.0>97.0, cone voltage of 45 V and collision energy of 25 V for the 4UA-2S-GlcNCOEt-6S internal standard.

The identity of the peaks for the KS disaccharides was confirmed by analysis of samples spiked with ^13^C-labelled g0A6 (Glycosyn, New Zeeland) and g6A6 (a kind gift from Biomarin, US) and for HS and DS disaccharides with samples spiked with unlabeled disaccharide solutions.

The concentration of the disaccharides was calculated using a calibration curve of each disaccharide with 4UA-2S-GlcNCOEt-6S (HD009, Iduron) as an internal standard. The following disaccharides were analyzed and used to calculate GAG concentrations: D0A0, D0S0, D0A6, D2A0, D0S6 and D2S0 (HS), D0a4 and D0a10 (DS), g0A6 and G6A6 (KS). All samples were digested and analyzed in triplicate.

Recovery of the analytes was determined in each experiment by spiking a control sample with heparan, dermatan and keratan sulfate (10 μg/mL each). Intra assay variation (coefficient of variation; CV) was determined for each GAG by repeated analysis (n = 10) of spiked and unspiked control samples and ranged from 9 to 18% for the individual GAGs. Inter-day CV was determined by replicate analysis of spiked high and low control samples on 10 separate days and was between 4–18%. The concentration of each GAG was linear at least up to 2 mg/mmol creat, which is twice the concentration observed in the most elevated patients samples.

### Statistical analysis

Statistical analysis was performed using SPSS Statistics software version 22 (IBM Corp., Armonk, NY, USA). Non-parametric ranking statistics (Mann-Whitney-U tests and Wilcoxon-rank test for unpaired and paired data respectively) were used to analyze the difference in GAG levels between groups.

**Fig 1:**
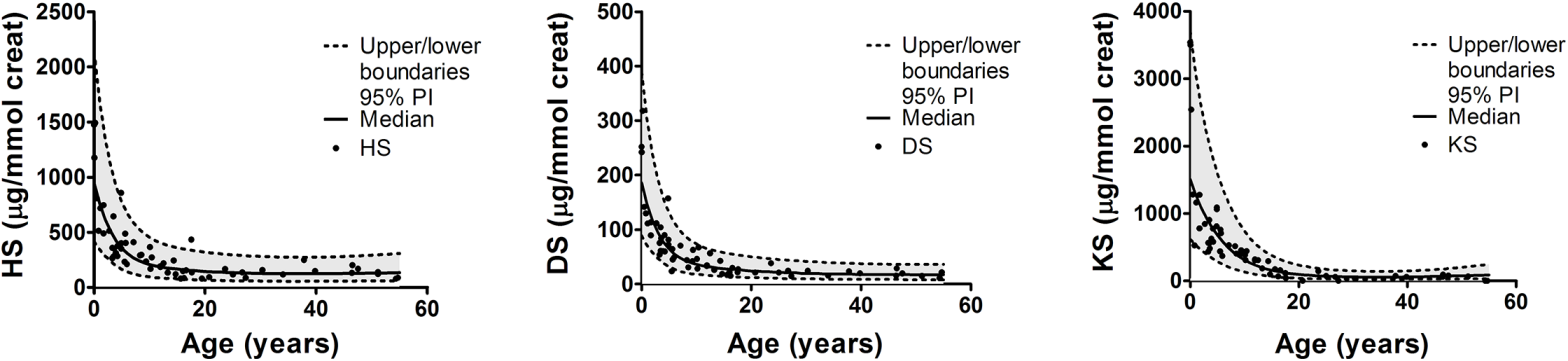
Modeled reference ranges for HS, DS, KS in urine. Individual data points of healthy controls are presented for HS, DS and KS as determined by the LC-MS/MS. Dashed lines indicate the boundaries of the 95% prediction interval. The upper limit of the 95% prediction interval is considered the upper limit of normal in this study.

Reference values were based on concentrations measured in healthy control samples. For HS, DS and KS, trends over age were fitted using restricted cubic splines. The upper limits of the 95% prediction interval for each one-year interval were considered the upper limit of normal (ULN). The ULN was calculated for HS, DS and KS separately, as well as the total of HS, DS and KS as analyzed by the LC-MS/MS method (Fig 1 and S1 Table). For the DMB, cut-off values as used in diagnostic daily practice were used as ULN (Table 3). For each measurement, the fold-ULN was calculated as follows:

$$\text{Fold-ULN}=\frac{\text{Observed GAG level}}{\text{Age-specific ULN}}$$

**Table 3:** Upper limit of normal for DMB per age category.

Age category	Upper limit of normal (mg/mmol creat)
**0–6 months**	50
**6–24 months**	20
**2–10 years**	15
**> 10 years**	8

The overall sensitivity and specificity were calculated for the LC-MS/MS assay and the DMB method for identification of MPS. The test was considered positive when one of the GAGs (LC-MS/MS) or the total GAG level (LC-MS/MS and DMB) was above the ULN. A p-value of <0.05 was considered statistically significant.

## Results

### GAG concentrations in MPS patients

**Fig 2:**
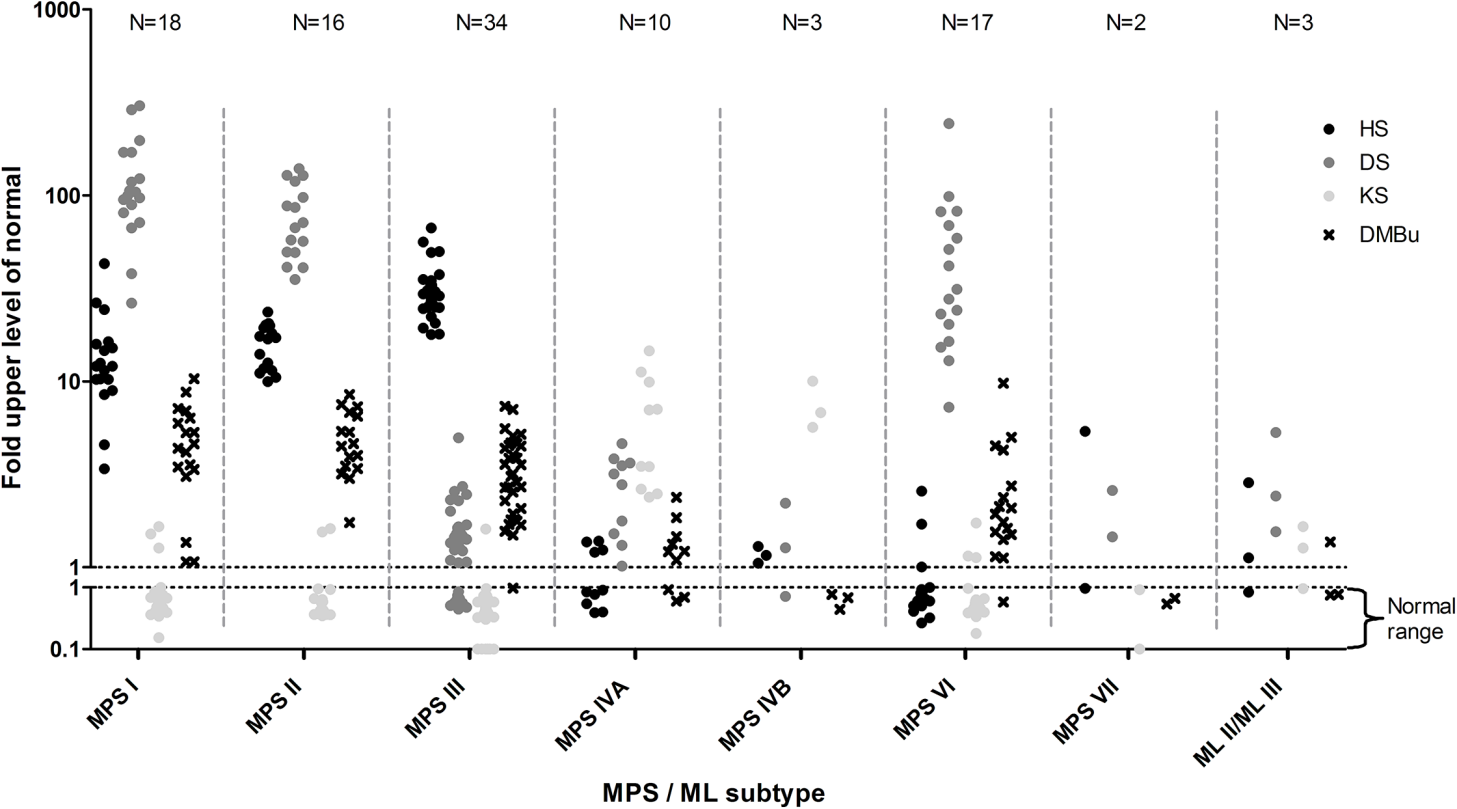
Concentrations of HS, DS and KS measured by multiplexed LC-MS/MS in confirmed MPS and ML patients. Values represent the fold-ULN.

Urine samples of 100 patients with a confirmed diagnosis of an MPS were analyzed by the LC-MS/MS assay and the DMB. The distribution of the patients, the age ranges per diagnosis and the median and range of the fold-ULN are presented in Table 2. Individual data points are depicted in Fig 2.

In all MPS I, MPS II, MPS III, MPS IVA, MPS IVB and MPS VI patients, an elevation of GAGs in a pattern that was in accordance with the underlying enzyme deficiency (Table 1) was detected by the multiplexed LC-MS/MS assay (Fig 2). In one MPS VII patient the DS concentration was only mildly elevated while the HS level was identical to the ULN. In the other MPS VII patient in this study an elevation of both HS and DS was detected.

**Fig 3:**
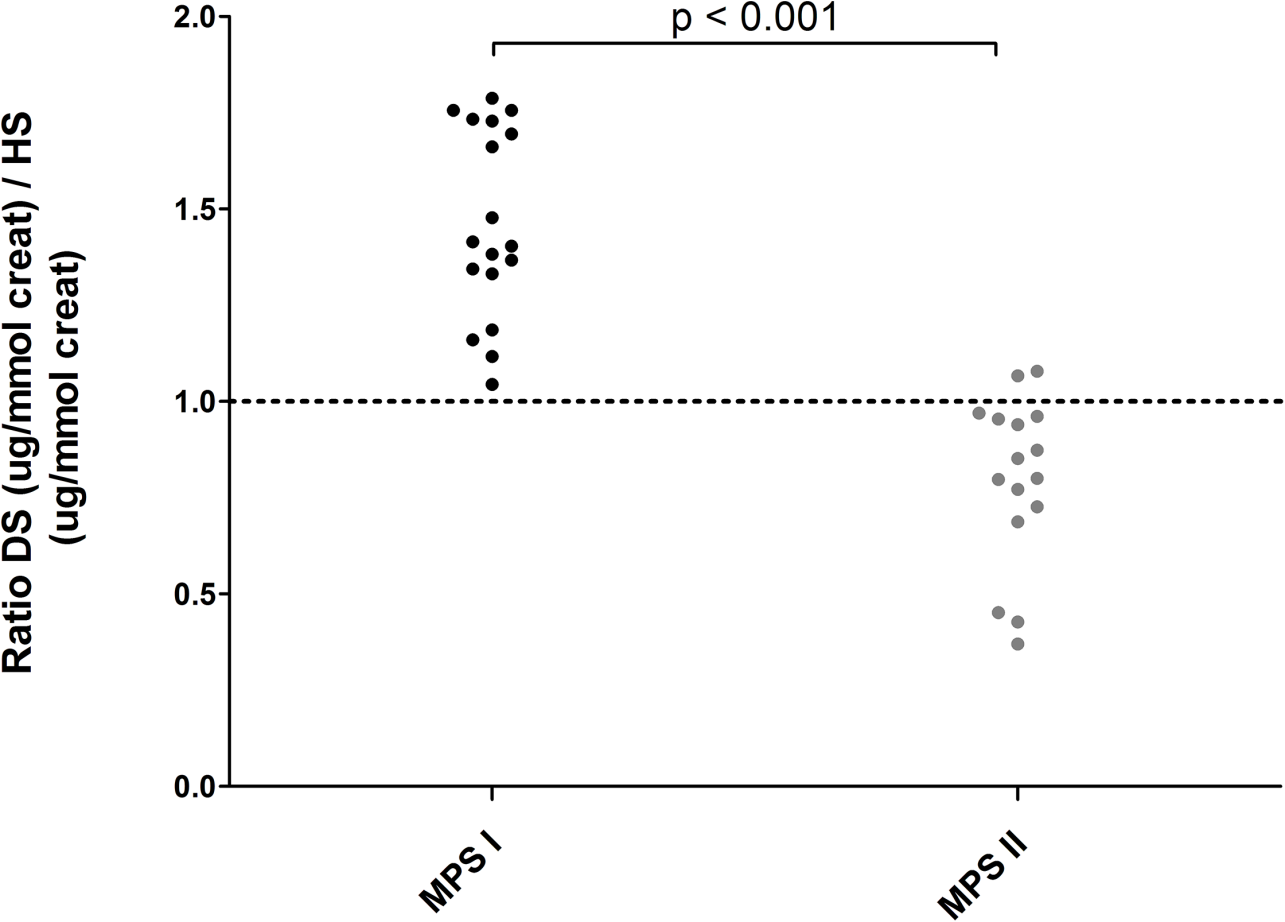
Ratio of the concentrations of DS and HS in MPS I and MPS II patients. In all MPS I patients a relatively high DS concentration (ratio >1) was observed, opposed to relatively higher HS levels (ratio <1) in MPS II patients.

HS and DS levels were elevated to similar levels in MPSI and MPSII patients (fold-ULN MPS I vs MPS II HS p = 0.11, DS p = 0.06). However, the ratio of absolute DS and HS levels differed significantly between the two diagnoses (DS:HS MPS I vs MPS II p<0.001). Although no absolute separation was observed, a ratio of <1.0 was only observed in MPS II patients (Fig 3).

Within each subtype, elevated levels of GAGs that are not a primary substrate of the deficient enzyme (Table 1) were detected in several patients. This secondary accumulation was low compared to the concentrations of the primary storage products, with a maximum fold-ULN of 4.9 seen in DS levels in one MPS III patient. Therefore, this secondary accumulation does not interfere with the discrimination of MPS subtypes based on the detected pattern of urinary GAG excretion.

**Fig 4:**
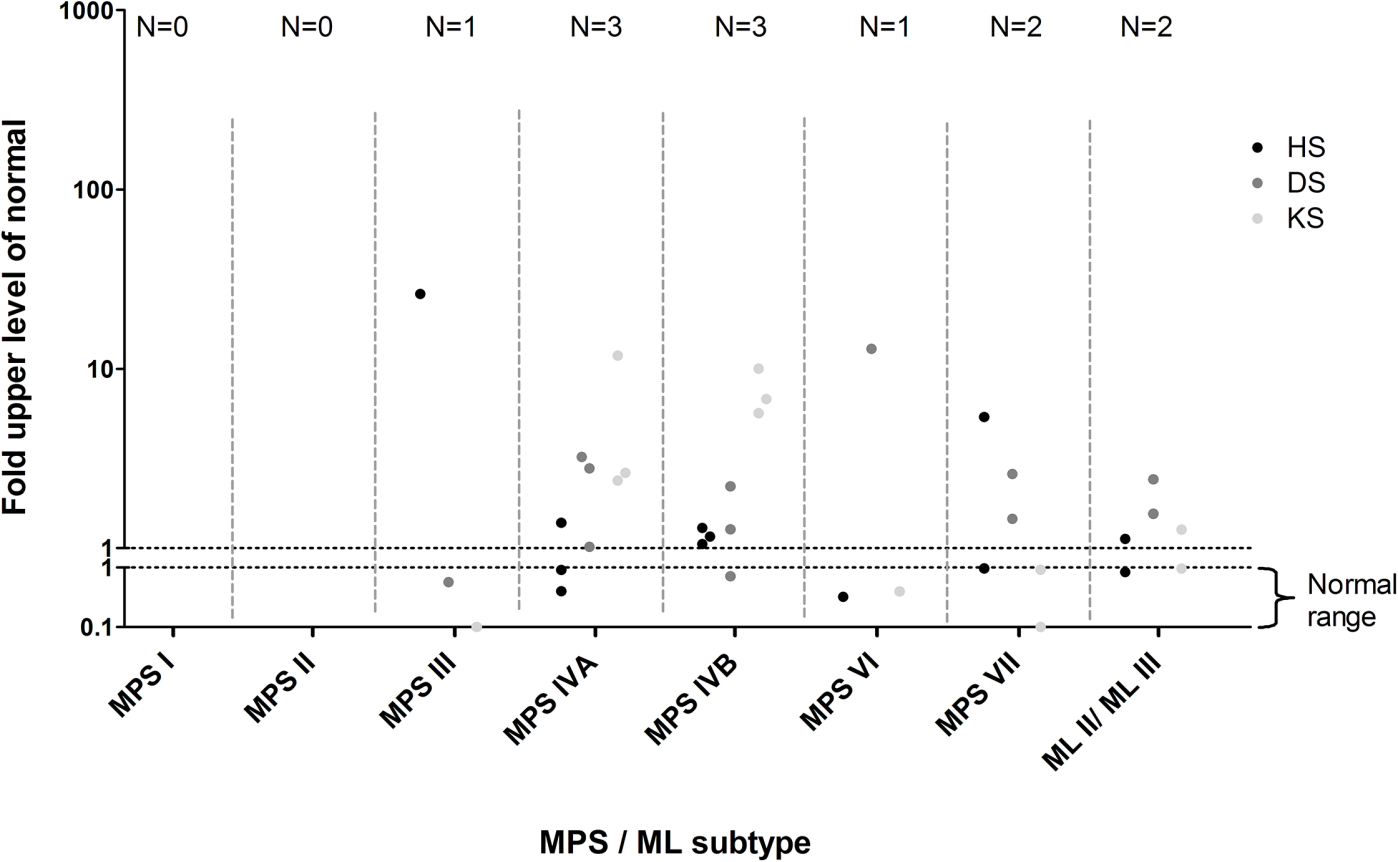
Concentrations of HS, DS and KS measured by multiplexed LC-MS/MS in confirmed MPS and ML patients with a DMB test within the normal range. Values represent the fold-ULN.

Of the 100 urine samples analyzed by DMB, 10 samples had urinary GAG levels within the normal range. In all these patients elevated GAGs could be detected by the LC-MS/MS method (Fig 4).

### GAG concentrations in ML patients

Both MLII patients had an abnormal GAG profile in urine. One had elevation of HS, DS and KS, while in the other only an elevation of DS was detected (Fig 2). In the MLIII patient studied mildly elevated levels of all three GAGs were observed. GAGs analyzed by DMB were within the normal range in one of the ML II patients and in the ML III patient (Fig 4).

### GAG concentrations in patients suspected for IEM

**Fig 5:**
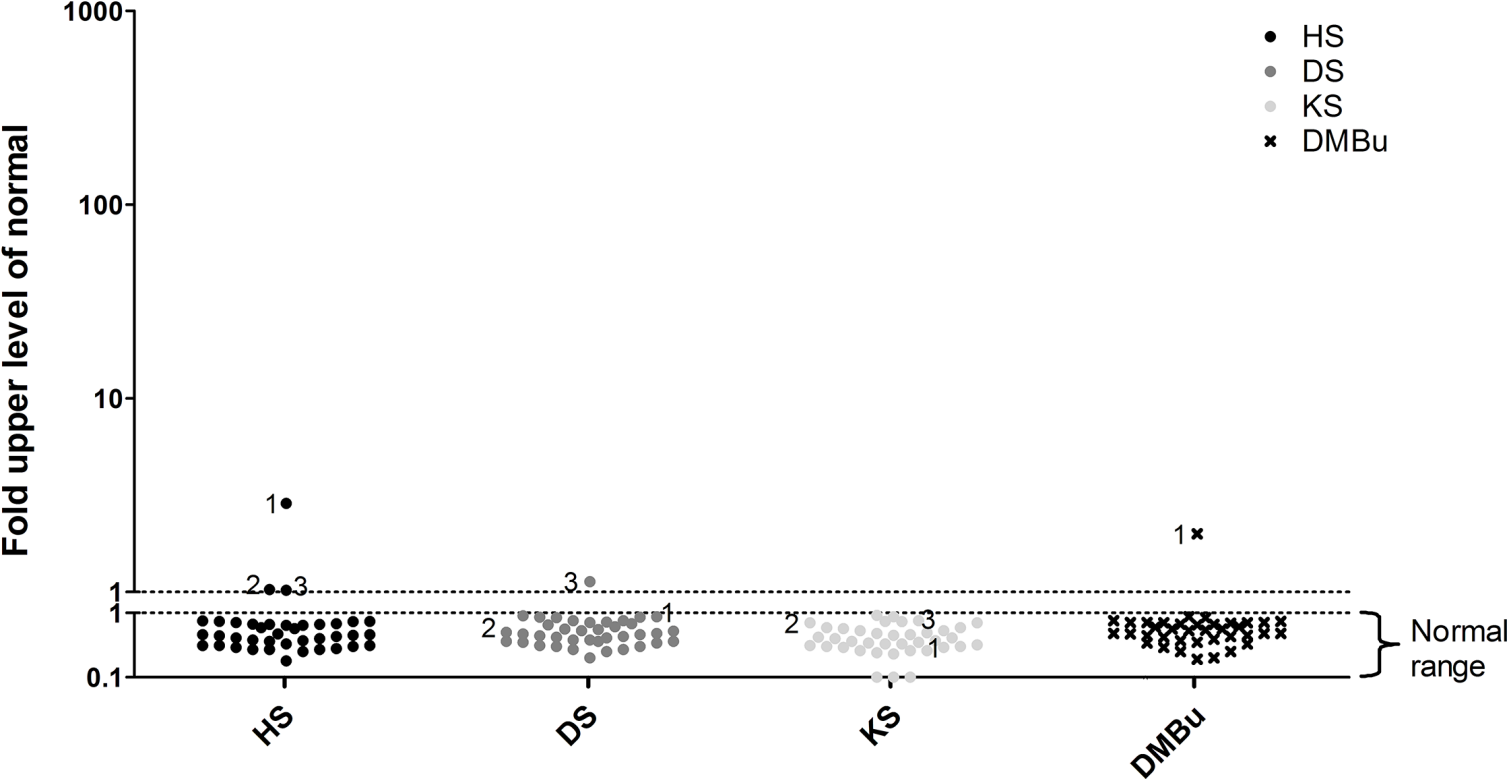
Concentrations of HS, DS, KS measured by multiplexed LC-MS/MS and total urinary GAGs by DMB in the patients suspected of an IEM. Urine samples of 39 patients with a suspected IEM with MPS in the differential diagnosis are included. Values represent fold-ULN. Patients that are considered false-positive are indicated by 1, 2 and 3.

Urine of thirty-nine patients suspected of an IEM, including MPSs, was analyzed by both the DMB and the multiplexed LC-MS/MS assay. The median age of these patients was 3.4 years (1 day – 62.5 years). Clinical characteristics included mental retardation (N = 20), motor development delay (N = 9), psychiatric symptoms (N = 8) and convulsions (N = 7), as well as dysmorphism, skeletal dysplasia and macrocephaly. In three of these 39 patients, an elevation of one or more GAGs was detected by the LC-MS/MS assay (Fig 5). Patient 1 had elevated HS, but not DS or KS. HS levels were well below the range observed in MPS III patients (2.9 fold-ULN versus 17.8–66.9 in MPS III patients). This sample was collected at the age of 9 months when the patient was on the ICU during a period of critical illness, including a resuscitation on the day of sample collection. The patient was previously diagnosed with a 22q11 deletion syndrome, complicated by an immunodeficiency and cardiac anomalies. In the urine sample of patient 2 elevated HS was detected as well, again well outside the range observed in MPS III patients (1.03 fold-ULN). This patient was a 5 days old neonate with cardiopulmonary insufficiency of unknown origin. He died one day after sample collection due to multi-organ failure. A causative diagnosis could not be made. In patient 3 marginally elevated HS (fold-ULN 1.02) and DS levels (fold-ULN 1.13) were observed at the age of 2 months. These GAG concentrations did not resemble the levels observed in any of the MPS/ML patients. This patient was a preterm infant in whom the post-natal period was complicated by necrotizing enterocolitis, unexplained cholestasis and a patent ductus arteriosus (PDA). By the age of six months the PDA and cholestasis were resolved. Based on the clinical characteristics, these patients are considered highly unlikely to have an MPS or ML, and are therefore considered false-positive. DMB GAG levels were within the normal range in 38 patients. (Fig 5). In one patient (patient 1) GAG levels were elevated. Electrophoresis was performed as part of the standard work up and the observed GAG pattern was normal.

### Overall test performance

GAGs were found to be elevated in all 100 MPS patients when analyzed by the LC-MS/MS assay. The true positive rate, or sensitivity, of the LC-MS/MS assay is therefore considered 100%. As 3 out of 39 patients suspected of an IEM had levels of >1 fold-ULN (false-positive), specificity of this test is calculated as 36/39 = 92%.

For the DMB assay, 10 out of the 100 MPS patients (1 MPS III, 3 MPS IVA, 3 MPS IVB, 1 MPS VI, 2 MPS VII,) had a value within the normal range. Sensitivity in this cohort was therefore 90/100 = 90%. For the cohort of patients suspected of an IEM one individual tested false-positive (patient 1) and a specificity of 38/39 = 97% was calculated.

No test performance was calculated for the ML patients due to the small sample size. Positive and negative predictive values could not be calculated, as the total of tested patients does not reflect the true incidence of MPS/ML in the population.

## Discussion

We show that the multiplexed LC-MS/MS assay reported here has excellent test characteristics for application as a first diagnostic test for all MPS subtypes. In a direct comparison with the currently widely used DMB test, the multiplexed LC-MS/MS method provides an important improvement in sensitivity (100% for the LC-MS/MS test vs 90% for the DMB).

Previous studies reported on the risk for false-negative DMB results in patients with MPS IVA [5]. Indeed, 3 out of the 10 MPS IVA patients in our study had a normal DMB value. Moreover, all three patients with MPS IVB had DMB levels within the normal range. While false-negative results are reported for MPS III patients for other dye binding assays, such as the Alcian blue based assay and the berry spot test [15,16], to our knowledge no false-negative DMB results have been reported in these patients. Our study reveals one MPS III patient with a normal DMB but a significantly increased HS level in urine. This patient has a very mild MPS IIIB phenotype with an IQ of 68 at the time of collection of the urine sample (at the age of 27 years). The diagnosis of attenuated MPS III is often complex, as initial presenting symptoms may be unspecific and somatic manifestations might be absent [17]. False-negative biochemical test-results in these patients may lead to a delayed diagnosis and missed opportunities for initiation of emerging treatments. We are not aware of any reports on patients with MPS VI with normal GAG excretion by DMB. However, 1 of the 17 patients with MPS VI in this study had a normal DMB test.

In current practice, quantification of GAGs by DMB is often followed by a time-consuming and semi-quantitative analysis of the GAG pattern by electrophoresis or thin layer chromatography. The LC-MS/MS assay, however, makes this second step redundant. Based on the observed pattern of GAG excretion, a direct identification of the MPS subtypes MPSIII, IV and VI is feasible. As expected, patients with MPS I and MPS II displayed a similar pattern of both HS and DS excretion and were indistinguishable based on the observed fold-ULN for these GAGs. However, the ratio of DS:HS was significantly different between MPS I and II, and similar observations can be extrapolated from data from other studies [7–9]. The relatively lower DS storage in MPS II might be due to the fact that iduronate-2-sulfatase (I2S) is only involved in the degradation of 2-sulfated iduronic acid. Although highly variable, the sulfated fraction of iduronic acid in DS is in general lower than in HS [18]. Consequently, in MPS II, DS degradation will be affected relatively less than HS degradation. In MPS I, however, α-L-iduronidase deficiency affects the degradation of all iduronic residues, rather than only the sulfated fraction, which may explain the different DS:HS ratio when compared to MPS II.

For MPS VII, an elevation of both HS and DS is expected, as β-glucuronidase is involved in the degradation of both GAGs. This was indeed observed in the urine of one of the two studied patients, while in the urine of the other patient an elevated DS was found but a HS concentration identical to the ULN. Relatively low levels of HS in MP VII have been reported previously [19]. Furthermore, both patients had DMB levels in the normal range.

Although GAG storage is observed in ML patients [20], DMB is often negative. This was confirmed in the three patients included in this study, as two had DMB levels within the normal range. However, all three ML II and ML III patients were identified by the LC-MS/MS assay. The fold-ULN in these patients was not as discriminatory as in MPS patients and further validation will be needed to establish the diagnostic value of the LC-MS/MS assay in mucolipidosis. However, our results underpin its potential as diagnostic tool for MPS related disorders that currently lack a practical diagnostic assay, which might also include multiple sulfatase deficiency and Sandhoff disease in addition to the mucolipidosis.

Our study has several limitations. First, the samples used for this study do not represent the distribution of MPS in the Dutch population [21], and the relative over-representation of MPS IVA and IVB patients may have led to the relatively low sensitivity observed for the DMB assay in this cohort. Second although sample selection was primarily based on availability of the material, a potential selection bias exists, where urine of MPS patients with borderline or normal DMB values in the primary diagnostic assay is more likely to be stored for future analysis by novel methods. This was indeed confirmed for two samples of patients with MPS VI in this study. Finally, only a small number of patients with MPS related disorders is included. The full diagnostic potential of the LC-MS/MS assay for these patients can therefore not be assessed.

In a number of patients, elevation of GAGs that are not a primary substrate of the deficient enzyme was detected. This phenomenon is known as secondary storage, and has previously been observed in MPS patients [22–24]. The secondary storage did, however, not interfere with the diagnostic potential of the assay.

This study focuses on the evaluation of the LC-MS/MS assay as a diagnostic test. In previous studies we showed that LC-MS/MS analysis of HS and DS in plasma and urine can be used for the evaluation of biochemical efficacy of enzyme replacement therapy in MPS I patients, also with characteristics superior to the use of DMB [13,25]. The multiplexed assay also includes KS analysis, and may therefore be applied in future studies as a sensitive tool for monitoring biochemical response to treatment in patients with various MPSs.

Three patients suspected of an IEM had an elevation of at least one GAG in the LC-MS/MS assay and these samples were collected during periods of critical illness. An increase in GAGs during an intercurrent illness in MPS patients has been observed by our group (unpublished data) and elevated GAG levels are also described in non-MPS diseases, such as rheumathoid arthritis (RA) and septic shock [26,27]. With the ULN based on the 95% prediction interval, a false-positive rate of 2.5% can be expected (type I error), but we show here that testing critically ill patients might lead to more false-positive results. Further studies will assess whether the ULN for the LC-MS/MS assay reported here can be optimized in order to increase the specificity for the diagnosis of MPSs and related disorders.

In summary, we provide evaluation of diagnostic accuracy of a straightforward assay that can be used for the diagnosis of the complete spectrum of MPS disorders and has the potential to identify patients with mucolipidosis II and III as well. The most prominent advantages of this multiplexed LC-MS/MS method are the superior sensitivity compared to the conventional DMB method, and the use of KS for the identification of MPS IVA and MPS IVB patients. In addition, the observed pattern of GAG elevation can accurately identify MPS subtypes, so there is no need for additional electrophoresis.

## Supporting Information

S1 Table Upper limit of normal references for heparan sulfate, dermatan sulfate, keratan sulfate and total HS, DS and KS in urine measured by multiplexed LC-MS/MS. ULN was based on the upper limit of the 95% prediction interval of a smooth curve, fitted on the data of 61 healthy controls.
(DOCX)
