# Supplementary material for: A Multiplex Assay for the Diagnosis of Mucopolysaccharidoses and Mucolipidoses
Source: PLoS One. 2015 Sep 25;10(9):e0138622. doi: 10.1371/journal.pone.0138622 (PMC4583541; doi:10.1371/journal.pone.0138622)
Supplement: S1 Table — ULN was based on the upper limit of the 95% prediction interval of a smooth curve, fitted on the data of 61 healthy controls. (DOCX) [file pone.0138622.s001.docx]

**Supplementary table 1. Upper limit of normal references for heparan sulfate, dermatan sulfate, keratan sulfate and total HS, DS and KS in urine measured by multiplexed LC-MS/MS.**ULN was based on the upper limit of the 95% prediction interval of a smooth curve, fitted on the data of 61 healthy controls.

|  | **Upper limit of normal (ULN)** | | | |
| --- | --- | --- | --- | --- |
| **Age (years)** | **Heparan sulfate (ug/mmol creat)** | **Dermatan sulfate (ug/mmol creat)** | **Keratan sulfate (ug/mmol creat)** | **Total HS, DS & KS (ug/mmol creat)** |
| **0** | 2138 | 389 | 3707 | 6971 |
| **1** | 1693 | 303 | 3093 | 5607 |
| **2** | 1356 | 239 | 2600 | 4554 |
| **3** | 1103 | 192 | 2201 | 3744 |
| **4** | 914 | 156 | 1874 | 3117 |
| **5** | 773 | 131 | 1601 | 2630 |
| **6** | 670 | 112 | 1369 | 2248 |
| **7** | 592 | 98 | 1170 | 1943 |
| **8** | 533 | 88 | 1001 | 1695 |
| **9** | 488 | 80 | 858 | 1492 |
| **10** | 452 | 74 | 737 | 1325 |
| **11** | 425 | 69 | 635 | 1187 |
| **12** | 403 | 65 | 551 | 1071 |
| **13** | 386 | 62 | 481 | 975 |
| **14** | 372 | 60 | 423 | 894 |
| **15** | 360 | 58 | 376 | 825 |
| **16** | 350 | 56 | 336 | 766 |
| **17** | 341 | 54 | 304 | 715 |
| **18** | 333 | 53 | 277 | 671 |
| **19** | 326 | 51 | 254 | 632 |
| **20** | 319 | 50 | 235 | 599 |
| **21** | 313 | 49 | 218 | 570 |
| **22** | 308 | 48 | 204 | 544 |
| **23** | 303 | 467 | 193 | 522 |
| **24** | 299 | 46 | 183 | 502 |
| **25** | 295 | 45 | 174 | 485 |
| **26** | 292 | 44 | 167 | 470 |
| **27** | 289 | 43 | 161 | 457 |
| **28** | 286 | 43 | 156 | 445 |
| **29** | 283 | 42 | 151 | 435 |
| **30** | 281 | 41 | 148 | 427 |
| **31** | 279 | 41 | 145 | 420 |
| **32** | 278 | 40 | 143 | 414 |
| **33** | 276 | 40 | 142 | 409 |
| **34** | 275 | 39 | 141 | 405 |
| **35** | 274 | 39 | 141 | 402 |
| **36** | 274 | 39 | 141 | 400 |
| **37** | 273 | 38 | 142 | 399 |
| **38** | 273 | 38 | 143 | 399 |
| **39** | 274 | 38 | 145 | 399 |
| **40** | 274 | 37 | 148 | 401 |
| **41** | 275 | 37 | 151 | 403 |
| **42** | 276 | 37 | 154 | 405 |
| **43** | 277 | 37 | 158 | 409 |
| **44** | 278 | 37 | 163 | 413 |
| **45** | 280 | 37 | 168 | 418 |
| **46** | 282 | 36 | 173 | 423 |
| **47** | 284 | 36 | 179 | 429 |
| **48** | 286 | 36 | 186 | 436 |
| **49** | 289 | 36 | 193 | 442 |
| **50** | 291 | 36 | 201 | 450 |
| **50+** | 294 | 36 | 209 | 458 |
